# Copper Amalgam

**Published:** 1888-08

**Authors:** 


ARTICLE V.

### COPPER AMALGAM.

The operator of today, who is not familiar with all the methods and materials known to dentistry, is certainly laboring at a disadvantage. The present tendency of American Dentistry is toward conservatism. We are learning to use more discrimination in the selection of filling materials, choosing those most suitable and serviceable for the case in hand, regardless of any previous prejudices which may have existed.

Copper amalgam is wholly different from other amalgams, in the fact that it contains nothing but pure copper, combined with mercury. This amalgam has been used in England for a great many years, but in this country it is almost unknown in its clinical aspect, and has not received the attention it merits.

It would seem at a glance that its use here had been confined more to experimental purposes, its general adoption as a filling material having been considerably neglected, and this, we think, quite unwisely. We have used copper amalgam quite extensively for several years (six or seven), and therefore can speak advisedly concerning the results obtained. These are sufficiently flattering to induce its continuance in the future, giving it the preference over other preparations bearing the stamp amalgam.

Our experience in the use of this material has been confined largely to the English preparation known as "Sullivan's Cement." The shrinking propensity, so common to the majority of amalgams now on the market, is almost unknown to the one under consideration. This alone is a most commendable feature. As a preserver of the teeth which fall under the head of faulty structure, this material has no equal. To further substantiate the above assertion, we take the liberty of quoting Dr. W. D. Miller, who says: "The only filling at present in use which exerts a continual anti-ferment action upon the walls of the tooth, and its immediate surroundings, is the old copper amalgam. Not only that, but the very substance of the tooth containing such a filling, itself becomes antiseptic. * * * Secondary decay in such a case would be next to impossible, where anything like cleanliness was observed. This result is well supported by observations which I have had abundant opportunity to make for the last five years, here where this material is so extensively used, and I do not hesitate to say that if our only object is to check the destruction of tissue by caries, there is no material at present in use with which this object may be so surely accomplished as with a good copper amalgam." Space forbids our commenting at this time upon the various theories regarding the manner in which this material preserves tooth structure; suffice it to say that chemists who may be considered as authorities, assert that this preservative property is the sulphide of copper, formed by the action of the saliva upon the metal, which has the power of preserving by its antiseptic virtues. Other observers claim that the copper salts which are formed, act purely in a mechanical manner, by plugging or sealing hermetically the dentinal tubuli, thus preventing the possibility of destructive agents penetrating, and acting upon the tooth in a locality protected by such a filling. The latter theory we regard as being quite plausible, and we do not wish to lose sight of the fact that the former is not valueless.

Objections have been raised against the use of copper amalgam, because it discolors the tooth. This is not universally true, as cases are seen where this material has been in use for five years, the teeth to all external appearances appearing as clear and perfect as the day they were filled. Again, other teeth have become discolored, but not to a degree to make them unsightly.

In those cases where discoloration had taken place, it was remembered that the amalgam, when mixed, was dirty, *i. e.*, there was a decided soiling of the hand or mortar in mixing; whereas, in the other cases, the amalgam left the hand unsoiled. We are not prepared to state positively that the salts of copper are self limiting in their penetration of dentine, but if this should be the case, we would not expect to find any discoration of the tooth.

We have lately been using some copper amalgam manufactured in Chicago, which for plasticity and general working qualities, is preferable to the English preparation; and the objectionable feature of uncleanliness seems to be entirely overcome by a new process in its manufacture. We therefore can safely predict that if the manufacturer uses care in its preparation, selecting his materials with a view of having them chemically pure, a copper amalgam can be obtained which will yield uniformly good results. And we need not fear that the tooth structure will be marred by discoloration, provided proper precautions are observed in its introduction.

The mechanical texture of copper amalgam is exceedingly compact, and the property it possesses of receiving the most delicate markings, makes it possible to adapt it to the inequalities of the cavity with but slight pressure. It would be imprudent to place it in a cavity exposed to view, because the surface of the filling turns black, and therefore would be unsightly. Where amalgam is to be used for filling temporary teeth, nothing better can be found than copper amalgam.

It is safe to predict that amalgams will not yet be dispensed with ; this being true, when we are compelled to use an amalgam, let it be the very best.—*The Dental Review.*

